# 3-Methyl-1-phenyl-4-[(phen­yl)(2-phenyl­hydrazin-1-yl)meth­ylidene]-1*H*-pyrazol-5(4*H*)-one

**DOI:** 10.1107/S1600536812013463

**Published:** 2012-04-04

**Authors:** Omoruyi G. Idemudia, Alexander P. Sadimenko, Anthony J. Afolayan, Eric C. Hosten

**Affiliations:** a University of Fort Hare, Department of Chemistry, Private Bag X1314, Alice 5700, South Africa; b Nelson Mandela Metropolitan University, Department of Chemistry, PO Box 77000, Port Elizabeth 6031, South Africa

## Abstract

The title compound, C_23_H_20_N_4_O, is a heterocyclic phenyl­hydrazone Schiff base with a pyrazole moiety. In the crystal, a variety of inter­actions occur, including N—H⋯π and π–π stacking between the phenyl ring of the phenyl­hydrazinyl group and its symmetry-generated equivalent [centroid–centroid distance = 3.6512 (7) Å].

## Related literature

For related structures, see: Zhu *et al.* (2010 ▶, 2011 ▶); Goh *et al.* (2009 ▶). For general background to pyrazolo­nes and their applications, see: Yang *et al.* (2000 ▶); Konstanti­novic *et al.* (2008 ▶); Joshi *et al.* (2011 ▶); Turan-Zitouni *et al.* (2000 ▶). For the biological activities of hydrazone Schiff bases, see: Yadav *et al.* (2010 ▶); Ozdemir *et al.* (2008 ▶); Vicini *et al.* (2006 ▶); Jagadeesh *et al.* (2010 ▶); Walcourt *et al.* (2004 ▶) and for their catalytic abilities, see: Pouralimardan *et al.* (2007 ▶).
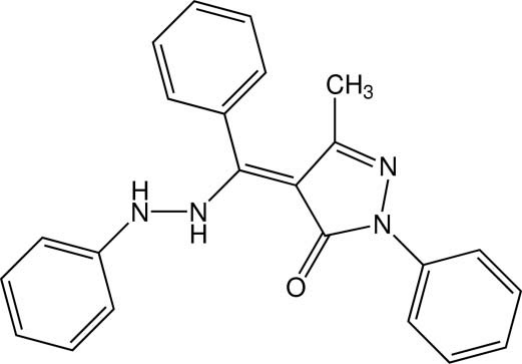


## Experimental

### 

#### Crystal data

C_23_H_20_N_4_O
*M* *_r_* = 368.43
Monoclinic, 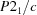

*a* = 8.6806 (2) Å
*b* = 20.4319 (4) Å
*c* = 10.6100 (2) Å
β = 99.713 (1)°
*V* = 1854.83 (7) Å^3^
*Z* = 4
Mo *K*α radiation
μ = 0.08 mm^−1^
*T* = 200 K
0.59 × 0.40 × 0.22 mm

#### Data collection

Bruker APEXII CCD diffractometer
Absorption correction: numerical (*SADABS*; Bruker, 2010 ▶) *T*_min_ = 0.87, *T*_max_ = 0.98
17965 measured reflections
4607 independent reflections
3891 reflections with *I* > 2σ(*I*)
*R*_int_ = 0.014

#### Refinement

*R*[*F* ^2^ > 2σ(*F* ^2^)] = 0.039
*wR*(*F* ^2^) = 0.108
*S* = 1.04
4607 reflections
262 parameters
H atoms treated by a mixture of independent and constrained refinement
Δρ_max_ = 0.27 e Å^−3^
Δρ_min_ = −0.17 e Å^−3^

### 

Data collection: *APEX2* (Bruker, 2010 ▶); cell refinement: *SAINT* (Bruker, 2010 ▶); data reduction: *SAINT*; program(s) used to solve structure: *SHELXS97* (Sheldrick, 2008 ▶); program(s) used to refine structure: *SHELXL97* (Sheldrick, 2008 ▶) and *ShelXle* (Hübschle *et al.*, 2011 ▶); molecular graphics: *ORTEP-3* (Farrugia,1997 ▶) and *Mercury* (Macrae *et al.*, 2008 ▶); software used to prepare material for publication: *PLATON* (Spek, 2009 ▶) and *publCIF* (Westrip, 2010 ▶).

## Supplementary Material

Crystal structure: contains datablock(s) global, I. DOI: 10.1107/S1600536812013463/zj2068sup1.cif

Structure factors: contains datablock(s) I. DOI: 10.1107/S1600536812013463/zj2068Isup2.hkl

Supplementary material file. DOI: 10.1107/S1600536812013463/zj2068Isup3.cdx

Supplementary material file. DOI: 10.1107/S1600536812013463/zj2068Isup4.cml

Additional supplementary materials:  crystallographic information; 3D view; checkCIF report

## Figures and Tables

**Table 1:** Hydrogen-bond geometry (Å, °) *Cg*2 is the centroid of the C11–C16 ring.

*D*—H⋯*A*	*D*—H	H⋯*A*	*D*⋯*A*	*D*—H⋯*A*
N4—H4⋯*Cg*2^i^	0.887 (18)	2.728 (17)	3.5021 (12)	146.6 (14)

Symmetry code: (i) 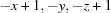.

**Fig. 1.**
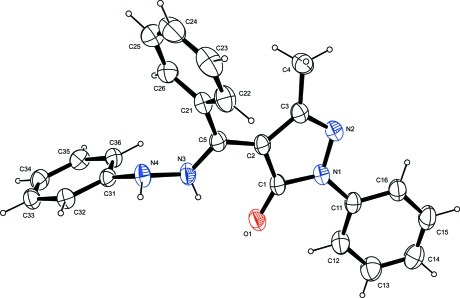
The molecular structure of the title compound, with atom labels and anistropic displacement ellipsoids (drawn at 50% probability level).

**Fig. 2.**
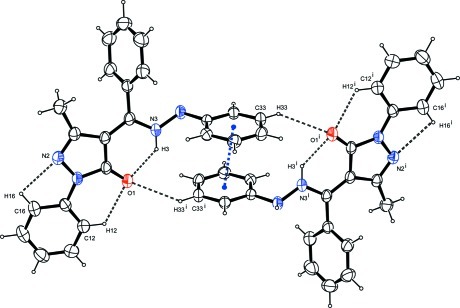
Inter and intra molecular contacts as well as the short ring interaction between C31—C36 and C31i—C36i (blue dashed line). Symmetry operators: i -x + 1, -y, -z + 2.

**Fig. 3.**
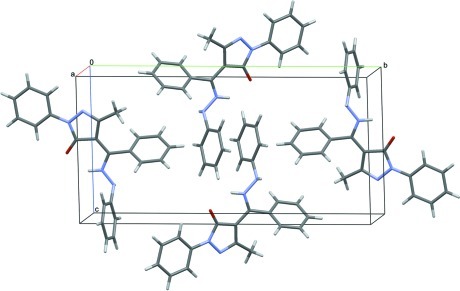
Molecular packing of the title compound, viewed along [1 0 0].

## supplementary crystallographic information

### Comment

Hydrazone Schiff bases, a product of the condensation reaction of hydrazine derivatives and a carbonyl *via* nucleophilic addition reaction represents an important group of compounds due to their chelating properties and numerous applications. Antimicrobial (Yadav *et al.*, 2010; Ozdemir *et al.*, 2008), antitumour (Vicini *et al.*, 2006), antioxidant (Jagadeesh *et al.*, 2010), antimalarial (Walcourt *et al.*, 2004) and catalytic (Pouralimardan *et al.*, 2007) activities, amidst others. Heterocyclic Schiff bases derived from pyrazolone have been well documented (Yang *et al.*, 2000). A new heterocyclic phenylhydrazone Schiff base with pyrazolyl moeity is prepared and its crystal structure reported herein (Fig.1).

In the crystal structure π stacking occurs between the phenyls of adjacent phenylhydazone groups with a centroid to centroid distance of 3.6512 (7) Å and slippage of 0.922 Å (Fig. 2). The dihedral angles formed by the least square planes between the phenyl of the phenylhydrazone group with the pyrazole and the C21—C26 aromatic ring are 85.29 (6)° and 77.88 (6)° repectively. The phenyl on the pyrazole group is slight twisted out of the pyrazole plane by 12.84 (4)°.

Intra molecular C—H···N, C—H···O and N—H···O interactions occur while inter molecular interactions include C—H···O, phenyl hydrazone pi stacking and N—H···π interactions (Fig. 2 - for clarity the N—H···π interaction is not shown).

The packing of the title compound is shown in Figure 3.

### Experimental

A mixture of phenylhydrazine and 4-benzoyl-3-methyl-1-phenyl-2-pyrazoline-5-one (ratio 1:1) in methanol was refluxed for 5 h. The mixture was poured into cold distilled water to precipitate the yellow titled compound (yield: 92%; m.p: 190–192°C), which was isolated by filtration and recrystalized from methanol. Single crystals of the titled compound suitable for X-ray diffraction was obtained from methanol by slow evaporation at room temperature.

### Refinement

The carbon-bound H atoms were placed in calculated positions (C—H 0.95 Å for aromatic carbon atoms and C—H 0.98 Å for the methyl group) and were included in the refinement in the riding model approximation, with *U*(H) set to 1.2*U*~eq~(C). The H atoms of the methyl group were allowed to rotate with a fixed angle around the C—C bond to best fit the experimental electron density (HFIX 137 in the *SHELX* program suite (Sheldrick, 2008), with U(H) set to 1.5 *U*~eq~(C)). The nitrogen-bound H atoms were located on a difference Fourier map and refined freely with isotropic parameters.

### Crystal data

**Table:**

C_23_H_20_N_4_O	*F*(000) = 776
*M**_r_* = 368.43	*D*_x_ = 1.319 Mg m^−^^3^
Monoclinic, *P*2_1_/*c*	Melting point: 464 K
Hall symbol: -P 2ybc	Mo *K*α radiation, λ = 0.71073 Å
*a* = 8.6806 (2) Å	Cell parameters from 200 reflections
*b* = 20.4319 (4) Å	θ = 2.6–26.6°
*c* = 10.6100 (2) Å	µ = 0.08 mm^−^^1^
β = 99.713 (1)°	*T* = 200 K
*V* = 1854.83 (7) Å^3^	Block, yellow
*Z* = 4	0.59 × 0.40 × 0.22 mm

### Data collection

**Table:**

Bruker APEXII CCD diffractometer	4607 independent reflections
Radiation source: sealed tube	3891 reflections with *I* > 2σ(*I*)
Graphite monochromator	*R*_int_ = 0.014
Detector resolution: 8.3333 pixels mm^-1^	θ_max_ = 28.3°, θ_min_ = 2.4°
φ and ω scans	*h* = −11→11
Absorption correction: numerical (*SADABS*; Bruker, 2010)	*k* = −27→26
*T*_min_ = 0.87, *T*_max_ = 0.98	*l* = −14→14
17965 measured reflections	

### Refinement

**Table:**

Refinement on *F*^2^	Primary atom site location: structure-invariant direct methods
Least-squares matrix: full	Secondary atom site location: difference Fourier map
*R*[*F*^2^ > 2σ(*F*^2^)] = 0.039	Hydrogen site location: inferred from neighbouring sites
*wR*(*F*^2^) = 0.108	H atoms treated by a mixture of independent and constrained refinement
*S* = 1.04	*w* = 1/[σ^2^(*F*_o_^2^) + (0.0506*P*)^2^ + 0.492*P*] where *P* = (*F*_o_^2^ + 2*F*_c_^2^)/3
4607 reflections	(Δ/σ)_max_ < 0.001
262 parameters	Δρ_max_ = 0.27 e Å^−^^3^
0 restraints	Δρ_min_ = −0.17 e Å^−^^3^

### Special details

**Table:**

Geometry. All e.s.d.'s (except the e.s.d. in the dihedral angle between two l.s. planes) are estimated using the full covariance matrix. The cell e.s.d.'s are taken into account individually in the estimation of e.s.d.'s in distances, angles and torsion angles; correlations between e.s.d.'s in cell parameters are only used when they are defined by crystal symmetry. An approximate (isotropic) treatment of cell e.s.d.'s is used for estimating e.s.d.'s involving l.s. planes.
Refinement. Refinement of *F*^2^ against ALL reflections. The weighted *R*-factor *wR* and goodness of fit *S* are based on *F*^2^, conventional *R*-factors *R* are based on *F*, with *F* set to zero for negative *F*^2^. The threshold expression of *F*^2^ > σ(*F*^2^) is used only for calculating *R*-factors(gt) *etc*. and is not relevant to the choice of reflections for refinement. *R*-factors based on *F*^2^ are statistically about twice as large as those based on *F*, and *R*- factors based on ALL data will be even larger.

### Fractional atomic coordinates and isotropic or equivalent isotropic displacement parameters (Å^2^)

**Table:**

	*x*	*y*	*z*	*U*_iso_*/*U*_eq_	
O1	0.37112 (10)	−0.06816 (4)	0.56481 (8)	0.0415 (2)	
N1	0.19594 (11)	−0.06784 (5)	0.37026 (8)	0.0317 (2)	
N2	0.08885 (10)	−0.02411 (5)	0.30147 (9)	0.0326 (2)	
N3	0.35993 (12)	0.04986 (5)	0.68161 (8)	0.0351 (2)	
H3	0.3866 (18)	0.0073 (8)	0.6855 (14)	0.049 (4)*	
N4	0.42974 (12)	0.09280 (5)	0.77706 (8)	0.0333 (2)	
H4	0.529 (2)	0.0996 (8)	0.7705 (16)	0.055 (4)*	
C1	0.27317 (13)	−0.04046 (6)	0.48275 (9)	0.0314 (2)	
C2	0.21562 (12)	0.02578 (5)	0.48023 (9)	0.0301 (2)	
C3	0.10127 (12)	0.03072 (5)	0.36586 (10)	0.0308 (2)	
C4	−0.00114 (14)	0.08688 (6)	0.31689 (12)	0.0410 (3)	
H4A	0.0612	0.1205	0.2829	0.062*	
H4B	−0.048	0.1054	0.3868	0.062*	
H4C	−0.084	0.0717	0.2488	0.062*	
C5	0.27016 (12)	0.07110 (5)	0.57521 (9)	0.0295 (2)	
C11	0.22094 (13)	−0.12928 (5)	0.31617 (10)	0.0318 (2)	
C12	0.31193 (16)	−0.17665 (6)	0.38711 (12)	0.0422 (3)	
H12	0.356	−0.1683	0.4738	0.051*	
C13	0.33855 (19)	−0.23605 (7)	0.33154 (14)	0.0521 (3)	
H13	0.4021	−0.268	0.3802	0.063*	
C14	0.27385 (18)	−0.24925 (7)	0.20636 (14)	0.0516 (3)	
H14	0.291	−0.2903	0.1692	0.062*	
C15	0.18389 (16)	−0.20201 (7)	0.13574 (12)	0.0477 (3)	
H15	0.1397	−0.2108	0.0493	0.057*	
C16	0.15679 (14)	−0.14194 (6)	0.18873 (11)	0.0390 (3)	
H16	0.0953	−0.1097	0.1389	0.047*	
C21	0.23894 (12)	0.14208 (5)	0.56126 (10)	0.0316 (2)	
C22	0.29871 (15)	0.17609 (6)	0.46630 (11)	0.0410 (3)	
H22	0.3529	0.1533	0.4092	0.049*	
C23	0.27886 (18)	0.24303 (7)	0.45546 (14)	0.0526 (4)	
H23	0.3216	0.2665	0.3922	0.063*	
C24	0.19696 (19)	0.27574 (7)	0.53651 (15)	0.0575 (4)	
H24	0.1834	0.3218	0.5289	0.069*	
C25	0.13434 (18)	0.24193 (7)	0.62892 (15)	0.0534 (4)	
H25	0.0757	0.2647	0.683	0.064*	
C26	0.15687 (14)	0.17513 (6)	0.64279 (12)	0.0401 (3)	
H26	0.1164	0.1521	0.7078	0.048*	
C31	0.40268 (12)	0.07932 (5)	0.90166 (9)	0.0275 (2)	
C32	0.51420 (13)	0.09944 (5)	1.00413 (10)	0.0314 (2)	
H32	0.6078	0.1197	0.989	0.038*	
C33	0.48897 (14)	0.08995 (6)	1.12813 (10)	0.0348 (2)	
H33	0.5649	0.1043	1.1978	0.042*	
C34	0.35439 (14)	0.05974 (6)	1.15146 (10)	0.0364 (2)	
H34	0.3384	0.0525	1.2368	0.044*	
C35	0.24276 (13)	0.04011 (6)	1.04931 (11)	0.0359 (2)	
H35	0.1497	0.0196	1.0651	0.043*	
C36	0.26506 (13)	0.05003 (5)	0.92422 (10)	0.0314 (2)	
H36	0.1873	0.037	0.8547	0.038*	

### Atomic displacement parameters (Å^2^)

**Table:**

	*U*^11^	*U*^22^	*U*^33^	*U*^12^	*U*^13^	*U*^23^
O1	0.0531 (5)	0.0408 (5)	0.0266 (4)	0.0052 (4)	−0.0043 (3)	−0.0013 (3)
N1	0.0352 (5)	0.0358 (5)	0.0233 (4)	−0.0036 (4)	0.0026 (3)	−0.0006 (3)
N2	0.0315 (4)	0.0386 (5)	0.0269 (4)	−0.0043 (4)	0.0028 (3)	0.0017 (4)
N3	0.0482 (6)	0.0343 (5)	0.0215 (4)	−0.0007 (4)	0.0023 (4)	−0.0021 (4)
N4	0.0386 (5)	0.0394 (5)	0.0217 (4)	−0.0079 (4)	0.0048 (4)	−0.0014 (4)
C1	0.0365 (5)	0.0374 (6)	0.0207 (4)	−0.0040 (4)	0.0056 (4)	−0.0008 (4)
C2	0.0332 (5)	0.0359 (5)	0.0218 (5)	−0.0031 (4)	0.0068 (4)	0.0012 (4)
C3	0.0295 (5)	0.0375 (6)	0.0261 (5)	−0.0053 (4)	0.0069 (4)	0.0019 (4)
C4	0.0387 (6)	0.0424 (6)	0.0394 (6)	0.0009 (5)	−0.0008 (5)	0.0016 (5)
C5	0.0316 (5)	0.0359 (5)	0.0224 (4)	−0.0033 (4)	0.0089 (4)	0.0011 (4)
C11	0.0338 (5)	0.0360 (5)	0.0268 (5)	−0.0089 (4)	0.0091 (4)	−0.0031 (4)
C12	0.0570 (7)	0.0376 (6)	0.0313 (6)	−0.0038 (5)	0.0048 (5)	−0.0010 (5)
C13	0.0703 (9)	0.0385 (7)	0.0465 (7)	0.0016 (6)	0.0069 (7)	−0.0017 (6)
C14	0.0616 (8)	0.0440 (7)	0.0502 (8)	−0.0049 (6)	0.0126 (6)	−0.0145 (6)
C15	0.0476 (7)	0.0586 (8)	0.0366 (6)	−0.0082 (6)	0.0065 (5)	−0.0175 (6)
C16	0.0371 (6)	0.0493 (7)	0.0304 (5)	−0.0048 (5)	0.0051 (4)	−0.0060 (5)
C21	0.0338 (5)	0.0342 (5)	0.0258 (5)	−0.0055 (4)	0.0022 (4)	−0.0003 (4)
C22	0.0480 (7)	0.0428 (7)	0.0318 (6)	−0.0101 (5)	0.0057 (5)	0.0034 (5)
C23	0.0622 (9)	0.0438 (7)	0.0465 (7)	−0.0173 (6)	−0.0060 (6)	0.0118 (6)
C24	0.0667 (9)	0.0340 (6)	0.0615 (9)	−0.0028 (6)	−0.0189 (7)	0.0021 (6)
C25	0.0573 (8)	0.0465 (7)	0.0519 (8)	0.0131 (6)	−0.0036 (6)	−0.0093 (6)
C26	0.0403 (6)	0.0444 (7)	0.0355 (6)	0.0027 (5)	0.0060 (5)	−0.0009 (5)
C31	0.0333 (5)	0.0267 (5)	0.0226 (4)	0.0014 (4)	0.0050 (4)	0.0004 (4)
C32	0.0326 (5)	0.0336 (5)	0.0276 (5)	−0.0032 (4)	0.0040 (4)	−0.0001 (4)
C33	0.0404 (6)	0.0378 (6)	0.0246 (5)	0.0008 (4)	0.0006 (4)	−0.0015 (4)
C34	0.0463 (6)	0.0387 (6)	0.0258 (5)	0.0029 (5)	0.0110 (4)	0.0029 (4)
C35	0.0374 (6)	0.0359 (6)	0.0367 (6)	−0.0030 (4)	0.0132 (4)	0.0021 (4)
C36	0.0334 (5)	0.0309 (5)	0.0293 (5)	−0.0029 (4)	0.0030 (4)	−0.0019 (4)

### Geometric parameters (Å, º)

**Table:**

O1—C1	1.2456 (13)	C15—C16	1.3865 (18)
N1—C1	1.3844 (13)	C15—H15	0.95
N1—N2	1.4021 (13)	C16—H16	0.95
N1—C11	1.4123 (14)	C21—C26	1.3856 (16)
N2—C3	1.3072 (14)	C21—C22	1.3941 (15)
N3—C5	1.3316 (14)	C22—C23	1.381 (2)
N3—N4	1.3983 (13)	C22—H22	0.95
N3—H3	0.899 (16)	C23—C24	1.378 (2)
N4—C31	1.4084 (13)	C23—H23	0.95
N4—H4	0.887 (17)	C24—C25	1.383 (2)
C1—C2	1.4414 (16)	C24—H24	0.95
C2—C5	1.3915 (15)	C25—C26	1.3832 (19)
C2—C3	1.4358 (14)	C25—H25	0.95
C3—C4	1.4903 (16)	C26—H26	0.95
C4—H4A	0.98	C31—C32	1.3903 (14)
C4—H4B	0.98	C31—C36	1.3925 (15)
C4—H4C	0.98	C32—C33	1.3834 (15)
C5—C21	1.4782 (15)	C32—H32	0.95
C11—C12	1.3880 (17)	C33—C34	1.3801 (17)
C11—C16	1.3968 (15)	C33—H33	0.95
C12—C13	1.3856 (19)	C34—C35	1.3858 (16)
C12—H12	0.95	C34—H34	0.95
C13—C14	1.379 (2)	C35—C36	1.3881 (15)
C13—H13	0.95	C35—H35	0.95
C14—C15	1.380 (2)	C36—H36	0.95
C14—H14	0.95		
			
C1—N1—N2	111.93 (9)	C14—C15—H15	119.4
C1—N1—C11	128.62 (9)	C16—C15—H15	119.4
N2—N1—C11	119.25 (9)	C15—C16—C11	119.29 (12)
C3—N2—N1	106.56 (9)	C15—C16—H16	120.4
C5—N3—N4	121.98 (10)	C11—C16—H16	120.4
C5—N3—H3	117.6 (10)	C26—C21—C22	120.10 (11)
N4—N3—H3	119.8 (10)	C26—C21—C5	121.36 (10)
N3—N4—C31	115.89 (9)	C22—C21—C5	118.50 (10)
N3—N4—H4	110.4 (11)	C23—C22—C21	119.83 (13)
C31—N4—H4	115.0 (11)	C23—C22—H22	120.1
O1—C1—N1	126.39 (11)	C21—C22—H22	120.1
O1—C1—C2	129.28 (10)	C24—C23—C22	119.92 (13)
N1—C1—C2	104.33 (9)	C24—C23—H23	120.0
C5—C2—C3	131.87 (10)	C22—C23—H23	120.0
C5—C2—C1	122.48 (10)	C23—C24—C25	120.39 (13)
C3—C2—C1	105.65 (9)	C23—C24—H24	119.8
N2—C3—C2	111.45 (10)	C25—C24—H24	119.8
N2—C3—C4	119.07 (10)	C26—C25—C24	120.19 (14)
C2—C3—C4	129.46 (10)	C26—C25—H25	119.9
C3—C4—H4A	109.5	C24—C25—H25	119.9
C3—C4—H4B	109.5	C25—C26—C21	119.52 (13)
H4A—C4—H4B	109.5	C25—C26—H26	120.2
C3—C4—H4C	109.5	C21—C26—H26	120.2
H4A—C4—H4C	109.5	C32—C31—C36	119.81 (9)
H4B—C4—H4C	109.5	C32—C31—N4	118.15 (9)
N3—C5—C2	118.43 (10)	C36—C31—N4	121.94 (9)
N3—C5—C21	118.41 (9)	C33—C32—C31	120.08 (10)
C2—C5—C21	123.10 (9)	C33—C32—H32	120.0
C12—C11—C16	119.55 (11)	C31—C32—H32	120.0
C12—C11—N1	120.79 (10)	C34—C33—C32	120.52 (10)
C16—C11—N1	119.64 (10)	C34—C33—H33	119.7
C13—C12—C11	120.03 (12)	C32—C33—H33	119.7
C13—C12—H12	120.0	C33—C34—C35	119.38 (10)
C11—C12—H12	120.0	C33—C34—H34	120.3
C14—C13—C12	120.75 (14)	C35—C34—H34	120.3
C14—C13—H13	119.6	C34—C35—C36	120.91 (10)
C12—C13—H13	119.6	C34—C35—H35	119.5
C13—C14—C15	119.17 (13)	C36—C35—H35	119.5
C13—C14—H14	120.4	C35—C36—C31	119.28 (10)
C15—C14—H14	120.4	C35—C36—H36	120.4
C14—C15—C16	121.20 (12)	C31—C36—H36	120.4
			
C1—N1—N2—C3	2.24 (12)	C11—C12—C13—C14	−0.9 (2)
C11—N1—N2—C3	−173.08 (9)	C12—C13—C14—C15	1.1 (2)
C5—N3—N4—C31	127.25 (11)	C13—C14—C15—C16	−0.5 (2)
N2—N1—C1—O1	177.77 (10)	C14—C15—C16—C11	−0.44 (19)
C11—N1—C1—O1	−7.46 (18)	C12—C11—C16—C15	0.69 (17)
N2—N1—C1—C2	−2.98 (11)	N1—C11—C16—C15	179.00 (10)
C11—N1—C1—C2	171.79 (10)	N3—C5—C21—C26	−64.01 (14)
O1—C1—C2—C5	2.00 (18)	C2—C5—C21—C26	118.77 (12)
N1—C1—C2—C5	−177.23 (9)	N3—C5—C21—C22	113.82 (12)
O1—C1—C2—C3	−178.25 (11)	C2—C5—C21—C22	−63.40 (14)
N1—C1—C2—C3	2.53 (11)	C26—C21—C22—C23	1.41 (18)
N1—N2—C3—C2	−0.47 (11)	C5—C21—C22—C23	−176.45 (11)
N1—N2—C3—C4	−179.01 (9)	C21—C22—C23—C24	−1.53 (19)
C5—C2—C3—N2	178.40 (10)	C22—C23—C24—C25	0.0 (2)
C1—C2—C3—N2	−1.33 (11)	C23—C24—C25—C26	1.6 (2)
C5—C2—C3—C4	−3.25 (19)	C24—C25—C26—C21	−1.7 (2)
C1—C2—C3—C4	177.03 (11)	C22—C21—C26—C25	0.22 (18)
N4—N3—C5—C2	174.74 (10)	C5—C21—C26—C25	178.02 (11)
N4—N3—C5—C21	−2.61 (15)	N3—N4—C31—C32	152.77 (10)
C3—C2—C5—N3	169.52 (11)	N3—N4—C31—C36	−30.79 (15)
C1—C2—C5—N3	−10.79 (15)	C36—C31—C32—C33	0.46 (16)
C3—C2—C5—C21	−13.26 (17)	N4—C31—C32—C33	176.98 (10)
C1—C2—C5—C21	166.43 (10)	C31—C32—C33—C34	0.88 (17)
C1—N1—C11—C12	13.08 (17)	C32—C33—C34—C35	−1.32 (18)
N2—N1—C11—C12	−172.48 (10)	C33—C34—C35—C36	0.44 (18)
C1—N1—C11—C16	−165.22 (11)	C34—C35—C36—C31	0.89 (17)
N2—N1—C11—C16	9.22 (15)	C32—C31—C36—C35	−1.33 (16)
C16—C11—C12—C13	−0.05 (19)	N4—C31—C36—C35	−177.71 (10)
N1—C11—C12—C13	−178.35 (12)		

### Hydrogen-bond geometry (Å, º)

Cg2 is the centroid of the C11–C16 ring.

**Table:**

*D*—H···*A*	*D*—H	H···*A*	*D*···*A*	*D*—H···*A*
N3—H3···O1	0.899 (16)	1.994 (16)	2.7204 (13)	136.9 (13)
C12—H12···O1	0.95	2.26	2.8993 (15)	124
C16—H16···N2	0.95	2.46	2.7950 (16)	100
C33—H33···O1^i^	0.95	2.59	3.3094 (13)	132
N4—H4···*Cg*2^ii^	0.887 (18)	2.728 (17)	3.5021 (12)	146.6 (14)

Symmetry codes: (i) −*x*+1, −*y*, −*z*+2; (ii) −*x*+1, −*y*, −*z*+1.
